# Efficacy of metamizole to prevent kidney injury after renal-ischaemic reperfusion injury in Wistar rats

**DOI:** 10.1097/MS9.0000000000001694

**Published:** 2024-01-12

**Authors:** Dhirajaya Dharma Kadar, Syah Mirsya Warli, Kiking Ritarwan, Muhammad Ichwan, Jufriady Ismi, Erjan Fikri, Juliandi Harahap, Ibnu Alferraly

**Affiliations:** a Division of Urology; b Division of Pediatric Surgery, Department of Surgery; c Department of Neurology, Faculty of Medicine, Universitas Sumatera Utara—Haji Adam Malik General Hospital; d Department of Urology, Universitas Sumatera Utara Hospital, Universitas Sumatera Utara; Departments of e Pharmacology and Therapeutics; f Public Health; g Anatomical Pathology, Faculty of Medicine, Universitas Sumatera Utara, Medan; h Department of Surgery, Faculty of Medicine, Universitas Syiah Kuala—Zainoel Abidin General Hospital, Banda Aceh, Indonesia

**Keywords:** EGTI, IL-18, metamizole, myeloperoxidase, NGAL, renal ischaemia-reperfusion injury

## Abstract

**Background:**

Renal ischaemia-reperfusion injury (RIRI) is a common kidney procedure complication due to temporary blood flow interruption, leading to kidney injuries. This study aimed to analyze the effect of metamizole on the levels of interleukin-18 (IL-18), neutrophil-gelatinase-associated lipocalin (NGAL), myeloperoxidase (MPO), and histopathological changes in rats with RIRI.

**Materials and methods:**

Animal pre-clinical design study was used. Thirty-two male Wistar rats (Rattus norvegicus) were divided into four groups: negative control, positive control, M100, and M200. Blood samples were collected by intracardiac puncture, followed by bilateral nephrectomy and analyzed histopathologically.

**Results:**

Significant difference in IL-18 levels between positive control vs negative control (114.1 + 12.07 vs. 94.0 + 11.4; *P* = 0.019) and positive control vs M100 (114.1 + 12.07 vs. 86.9 + 8.34; *P* = 0.007). There was no difference in NGAL. M100 group had the lowest serum MPO levels (14.78+2.01), there was a significant difference in MPO levels in all pairwise analyses. There was a difference in cumulative EGTI scores among the study groups [positive 10.5 (8–11) vs. negative 9 (7–10) vs. M100 9 (7–10) vs. M200 9 (7–11); *P* = 0.021].

**Conclusion:**

Metamizole 100 mg/kgBW can reduce IL-18 and MPO levels in RIRI, giving more optimal results without affecting NGAL levels. Metamizole administration can reduce cumulative EGTI scores in RIRI, both at doses of 100 mg/kgBW and 200 mg/kgBW. This study shows that Metamizole can be used to prevent kidney injury caused by RIRI. IL-18 and MPO can be biomarkers in predicting kidney injury in RIRI.

## Introduction

Highlights

- Metamizole has the potential to serve as a renal protective agent in the context of renal ischaemia-reperfusion injury (RIRI).
- Metamizole administration resulted in a noteworthy decrease in interleukin-18 levels and myeloperoxidase levels in RIRI, with the most effective dosage identified was 100 mg/kg body weight.
- Metamizole did not showed and promise in lowering interleukin-18 and neutrophil-gelatinase-associated lipocalin levels during RIRI.
- Metamizole demonstrated its ability to reduce histopathological kidney damage, as evidenced by a decrease in the cumulative score of the endothelial, glomerular, tubular, and interstitial.

Renal ischaemia-reperfusion injury (RIRI) is a common complication of kidney procedures that involves temporary interruption of blood flow, such as transplantation or nephrectomy^1^. RIRI occurs when blood flow is restored to the kidney after a period of ischaemia, causing oxidative stress and inflammation. The mechanism of RIRI is the increase of polymorphonuclear cell activity at the site of injury after the restoration of blood flow to the kidney^1^. These cells will release excessive inflammatory mediators such as myeloperoxidase (MPO), which will trigger the formation of hypochlorous acid (HOCl). Hypochlorous acid can cause tissue damage through its interaction with amino acids, fats, and nucleic acids in body cells^1^. RIRI is especially a concern for urologic surgeons who often require procedures that induce ischaemic kidney events in nephron-sparing surgery to minimize bleeding^2^.

RIRI can lead to acute kidney injury (AKI) and chronic kidney disease (CKD) if not prevented or treated^3^. AKI is defined as a rapid decrease in glomerular filtration rate (GFR) that occurs over hours or days^3^. The newly developed RIFLE criteria have attempted to simplify the clinical definition and classification of AKI^4^. Biomarkers that are released into the blood or urine by damaged kidneys, similar to the release of troponin by injured myocardial cells after myocardial ischaemia, may be more sensitive and specific markers of AKI than serum urea and serum creatinine^3^. In addition, early detection of AKI with kidney-specific biomarkers may result in earlier nephrology consultation, more optimal antibiotic dosing, avoidance of nephrotoxic agents and even early specific therapy to repair damaged kidneys^3^. The ideal AKI biomarker would enable early detection of kidney injury before serum creatinine and/or serum urea increase; would differentiate acute tubular necrosis (ATN) from acute glomerulonephritis or acute interstitial nephritis; would be able to monitor the effects of intervention or treatment; and would predict the need for dialysis, death, and long-term kidney outcome^3^. Some biomarkers that can be used as markers of AKI condition include interleukin-18 (IL-18)^5^, neutrophil-gelatinase-associated lipocalin (NGAL)^6–8^, and myeloperoxidase (MPO)^9^.

RIRI is also a complex process where histopathologically there is damage to the tubular cells, endothelial cells, glomerular cells and tubulo-interstitial cells^10^. Typical events that mark RIRI include loss of endothelial cell integrity, acute tubular necrosis, tubulo-interstitial cell damage and ischaemic damage to glomerular cells^10^. A study by Khalid *et al.* ^10^ in 2016 showed that the endothelial, glomerular, tubular, and interstitial (EGTI) scoring system provides a more comprehensive assessment of kidney injury due to RIRI where it was found that the EGTI scoring system correlated significantly with serum creatinine and also AKI markers such as NGAL and KIM-1.

Recent literatures have found an inhibitory effect of metamizole, a popular non-opioid analgetic, on MPO (a crucial inflammatory derivative that is involved in RIRI) when given in a specific dosage^11^. Reperfusion injury may occur as polymorphonuclear leucocyte (PNL) produce high number of MPO, elevating toxic HOCI level. Inhibition by Metamizole on the preliminary cascading process may be clinically beneficial, albeit very limited evidence is currently available in literatures^12^. Furthermore, a study by Kumbasar and colleagues suggested that the use of metamizole could prevent Ischaemia-Reperfusion Injury in ovarian tissues. However, no research has assessed its impact on kidney tissues. Therefore, we aims to evaluate the influence of metamizole administration in preventing RIRI in Wistar rats, utilizing histological examinations for assessment. Therefore, this study aimed to analyze the effect of metamizole on the levels of IL-18, NGAL, MPO, and histopathological changes in rats with RIRI.

## Methods

### Animal preparation

This study used an animal pre-clinical design and have been reported in line with the ARRIVE criteria, using Wistar rats^13^. Thirty-two male Wistar rats (*Rattus norvegicus*) were divided into four groups: negative control, positive control, group treated with metamizole 100 mg/kgBW, and group treated with metamizole 200 mg/kgBW. The inclusion criteria in this study were male rats (to avoid hormonal influences on the results of the study) and weighed between 200 and 250 g. Wistar rats were preferred due to its high availability, low maintenance, and high versatility for most experimental study. Wistar rats is known to be well extrapolated to be applied on humans. Inclusion was strictly for male due to hormonal influences in female Wistar rats^13^. The exclusion criteria in this study were rats that had macroscopic kidney disorders found during kidney clamping. The dose of 100 mg/kgBW was chosen because that dose is the standard first dose for administering metamizole therapy to prevent reperfusion injury. The dose of 200 mg/kgBW was chosen as a comparator because that dose has been proven effective in preventing reperfusion injury in other organs, evidenced by the low level of Myeloperoxidase in organs given intervention in the form of metamizole 200 mg/kgBW^14^. Metamizole was injected into rats with the third and fourth groups, then waited for 30 min before proceeding into the surgical procedure. Research approval was obtained from the Health Research Ethics Committee of USU Faculty of Medicine with Number 1193/KEP/USU/2021.

### Sample calculation

We estimated the sample size using the Federer formula:


(n−1)(t−1)≥15

n= samples per study group

t= total of study group

Since we planned to have four group of rats in this study, we calculated the formula and resulted in 6 minimum rat samples per group, and 24 total samples of mice. All mice were appointed randomly to each group.

### Surgical procedure

All rats undergone surgery twice. First procedure is to open the abdomen using midline incision and manipulation were divided into two, one only manipulating the intraperitoneal organs, and one to clamp the bilateral kidneys. The second procedure is to do another open surgery twelve hours after the previous manipulation for blood and histopathological sample collections. The first procedure (manipulation) and second procedure (sample collections) were done by two different surgeons to blind the study. The second surgeon who did the sample collections were blinded, not knowing which rats belong to which group. All the rats were randomized

In the first group, after the abdomen was opened, manipulation was performed on the intraperitoneal organs. Then proceeded with suturing of the abdominal wall. Then the rats were returned to the cage.

In the second group, third group and fourth group, after the abdomen was opened, identification of both renal pedicles was performed, then clamping was performed on both renal pedicles (bilateral clamping technique) using Bulldog Clamp brand Renz for 45 min then both clamps were removed, abdomen was closed and rats were returned to cage.

**Figure 1:**
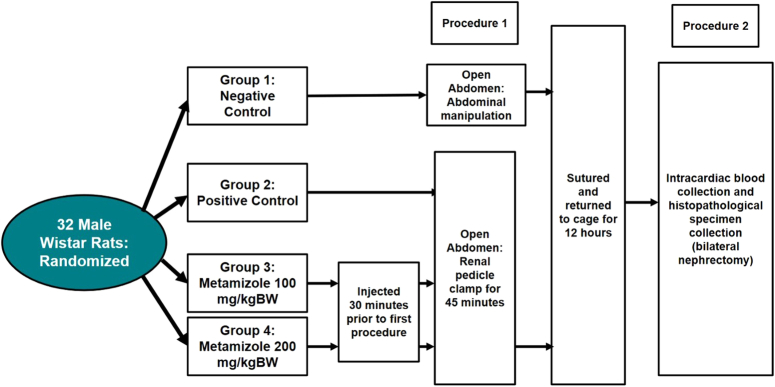
Experimental flow chart.

Twelve hours after being put in a cage, all of the rats would undergone intracardial blood sampling and nephrectomy of both kidneys. Detailed process is explained in Figure 1.

### Blood and kidney sample preparation

The first sample preparation was blood collection. Twelve hours after the manipulation, all groups were prepared for blood sampling and 3 ml of blood was taken intracardially. Intracardial blood withdrawal was preferred due to the large amount of blood needed (in comparison to the weight of each mouse, which are ~200–250 g) and samples were non survival afterwards. The blood sample was then put into an EDTA tube and centrifuged for 10 min at a rotation speed of 3000 rpm. After blood components were separated, supernatant fluid (serum) was taken to examine the IL-18, NGAL and MPO levels (Merck Sigma Aldrich ELISA Kit).

The second sample preparation was the histopathological examination from the resected bilateral kidneys. In histopathological examination, each kidney sample was immersed in 4% formaldehyde solution and fixed with paraffin block. Sections through the kidney were stained with hematoxylin-eosin and preparations were examined using a light microscope and assessed based on EGTI scoring.

Sample treatments on experimental animals were carried out at the Pharmacology Laboratory of USU Faculty of Medicine and the examination of IL-18, NGAL, and Myeloperoxidase levels was carried out at the Integrated Laboratory of USU Faculty of Medicine. Histopathological examination was performed at the Department of Anatomical Pathology of USU Faculty of Medicine. The study was conducted from August 2021 to January 2023.

### Statistical analysis

Outcome variables, namely IL-8, NGAL, and MPO, are presented as continuous outcome as mean ± standard deviation. Comparison between each group were done using ANOVA test, and post-hoc Bonferroni test was then performed to assess the differences between the four groups pairwise. Statistical significance is defined as *P* valueless than 0.05. All statistical analysis was done using IBM SPSS ver 23 (IBM, Copenhagen).

## Results

**Figure 2:**
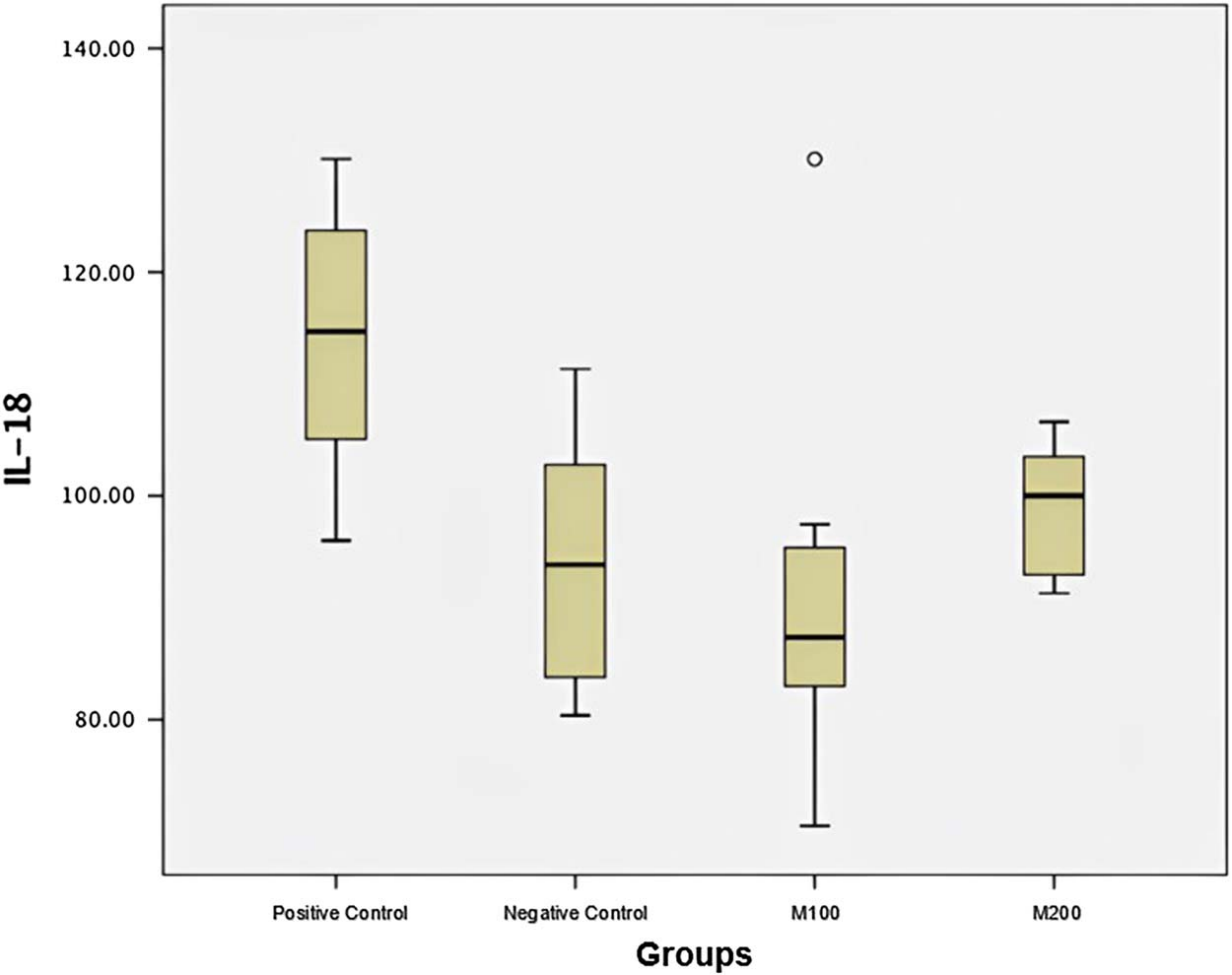
Box plot of interleukin-18 (IL-18) level.

There was a significant difference in IL-18 levels between positive control vs. negative control (114.1 + 12.07 vs. 94.0 + 11.4; *P* = 0.019) and positive control vs. M100 (114.1 + 12.07 vs. 86.9 + 8.34; *P* = 0.007) (Table 1 and Fig. 2).

**Table 1:** Interleukin-18 level in all groups

Variable	Positive control	Negative control	M100	M200	*p* a
IL-18	114.1+12.07	94.0+11.4	86.9+8.34	98.8+5.83	<0.001

IL-18, interleukin-18.

a One-way ANOVA test.

Due to the normal distribution of data, parametric testing was performed using One Way ANOVA. It was found that the sample that received M100 intervention had an IL-18 level of 86.9 + 8.34, which was the lowest among the other groups (Table 2). Post-Hoc Bonferroni test was then performed to assess the differences between the four groups pairwise, and it was found that there were significant differences in IL-18 levels between positive control vs. negative control and positive control vs M100 (Table 2).

**Table 2:** Post-hoc analysis of interleukin-18 level

Variable	Group		*p* a	Post-hoc analysis	*P* b
IL-18	Positive control	114.1+12.07	<0,001	Positive control vs. negative control	0.019
				Positive control vs. M100	0.007
				Positive control vs. M200	0.123
	Negative control	94.0+11.4		Negative control vs positive control	0.019
				Negative control vs. M100	1.000
				Negative control vs. M200	1.000
	M100	86.9+8.34		M100 vs. positive control	0.007
				M100 vs. negative control	1.000
				M100 vs. M200	1.000
	M200	98.8+5.83		M200 vs. positive control	0.123
				M200 vs. negative control	1.000
				M200 vs. M100	1.000

IL-18, interleukin-18.

a One-way ANOVA test.

b Post-hoc Bonferroni test.

**Figure 3:**
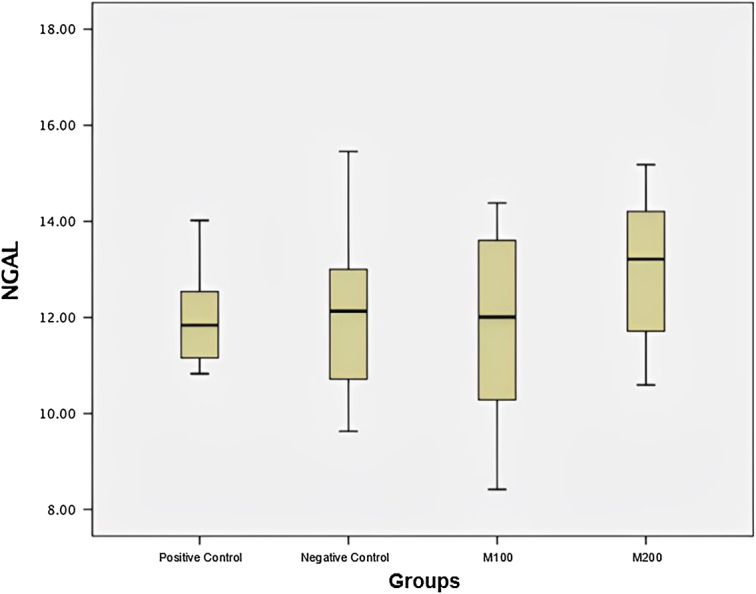
Box plot of neutrophil-gelatinase-associated lipocalin (NGAL) level.

There was no difference in NGAL among the study groups. The M200 group had an average NGAL value of 13.0+1.59 as the group with the highest level, while the positive control group had an average NGAL of 11.99+1.06 and was the group with the lowest level (Table 3 and Fig. 3).

**Table 3:** Neutrophil-gelatinase-associated lipocalin level in all groups

Variable	Positive control	Negative control	M100	M200	*p* a
NGAL	11.99+1.06	12.09+1.85	12.75+4.11	13.0+1.59	0.805

NGAL, neutrophil-gelatinase-associated lipocalin.

a One-way ANOVA test.

**Figure 4:**
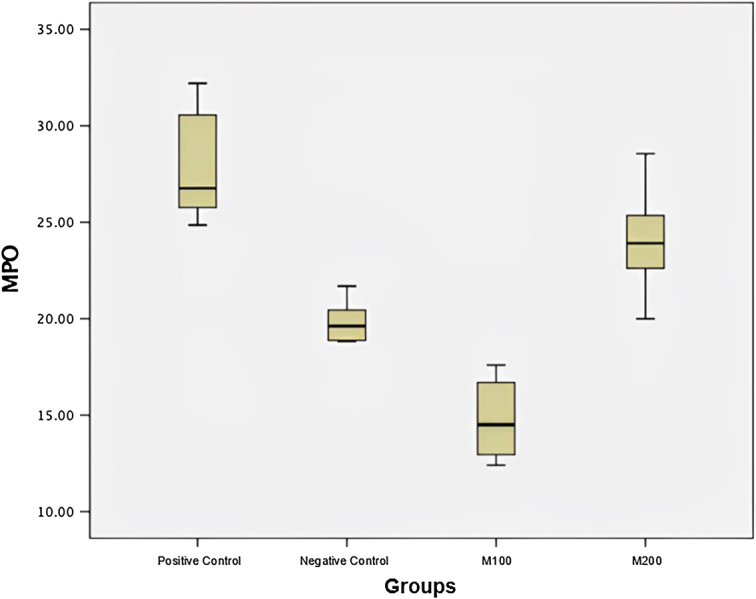
Box plot of myeloperoxidase (MPO) level.

Samples that received M100 intervention had the lowest serum MPO levels compared to other groups (14.78+2.01), and there was a significant difference in MPO levels in all pairwise analyses (Table 4, Fig. 4, and Table 5).

**Table 4:** Myeloperoxidase level in all groups

Variable	Positive control	Negative control	M100	M200	*p* a
MPO	27.,90+2.85	19.79+1.01	14.78+2.01	24.04+2.54	<0.001

MPO, myeloperoxidase.

a One-way ANOVA test.

**Table 5:** Post-hoc analysis of myeloperoxidase level

Variable	Group		*p* a	Post-hoc analysis	*P* b
MPO	Positive control	27.90+2.85	<0.001	Positive control vs. negative control	<0.001
				Positive control vs. M100	<0.001
				Positive control vs. M200	0.010
	Negative control	19.79+1.01		Negative control vs positive control	<0.001
				Negative control vs. M100	0.001
				Negative control vs. M200	0.004
	M100	14.78+2.01		M100 vs. positive control	<0.001
				M100 vs. negative control	0.001
				M100 vs. M200	<0.001
	M200	24.04+2.54		M200 vs. positive control	0.010
				M200 vs. negative control	0.004
				M200 vs. M100	<0.001

MPO, myeloperoxidase.

a One-way ANOVA test.

b Post-hoc Bonferroni test.

There was a difference in cumulative EGTI scores among the study groups [positive 10.5 (8–11) vs. negative 9 (7–10) vs. M100 9 (7–10) vs. M200 9 (7–11); *P* = 0.021) (Table 6).

**Table 6:** Endothelial, glomerular, tubular, and interstitial score

Variable	Positive control	Negative control	M100	M200	*p* a
EGTI	10.5 (8–11)	9 (7–10)	9 (7–10)	9 (7–11)	0.021

EGTI, endothelial, glomerular, tubular, and interstitial.

a Kruskal–Wallis test.

## Discussion

Kidney injury associated with ischaemia/reperfusion results from a dynamic process involving inflammation and several mediators in complex interactions. The formation of oxidative stress and lipid peroxidation seems to be a major factor driving the inflammatory process during RIRI^15^. A better understanding of the pathophysiology and therapeutic approaches underlying the functional defects found in ischaemic acute kidney failure will also require us to remember the complexity of the disease^15^.

From the results of this study, it was found that administration of metamizole 100 mg/kgBW resulted in lower IL-18 levels compared to positive control and rat group given metamizole 200 mg/kgBW. In accordance with the study by Steuber and colleagues who found that in patients given metamizole doses with minimal toxicity dose, no increase in AKI incidence was found compared to patients who did not receive metamizole. Whereas if given a dose that exceeds the minimal toxicity dose, a significant increase in AKI incidence was found. Every 1 g increase of metamizole increases the risk of drug-induced nephropathy by 1.6×. This can be explained through the mechanism of action of metamizole which blocks the synthesis of prostaglandin E2. Under conditions when the neurohormonal system is activated, prostaglandin E2 acts as a vasodilator that maintains blood flow to the kidney. Therefore, it is estimated that at a dose of 200 mg/kgBW has passed the minimal toxicity dose. Therefore, it can be concluded that administration of Metamizole 100 mg/kgBW gives optimal results to prevent RIRI events in IL-18 parameters.

In this study, there was no significant difference in NGAL levels between the study groups. Studies that explain the relationship between metamizole and NGAL have not been done much. Schulte *et al.* ^15^. showed that metamizole administration did not yield significant results, although higher levels were found compared to the group that did not receive metamizole. In that study, it was mentioned that metamizole was associated with high NGAL levels and NGAL was considered an early marker of kidney injury even at subclinical levels^15^. The insignificant results in NGAL levels were estimated to be due to several reasons, among others: (1) studies related to increased NGAL at 12–48 h post RIRI were conducted on mice while this study was conducted on Wistar rats, where it is suspected that increased NGAL levels require longer time; (2) In addition to being produced by injured renal tubules, NGAL can also be produced by neutrophils, which can cause bias that results in NGAL being nonspecific to RIRI events; (3) The reagents used to measure NGAL levels are currently unable to distinguish NGAL produced by renal tubules or by neutrophils^16^.

In this study, different levels of MPO were obtained among the research groups. It was found that the highest mean MPO was in the positive control experimental rats (27.90+2.85) and the lowest MPO level was in the research group that received metamizole 100 mg/kgBW (14.78+2.01). When a pairwise post-hoc analysis was performed, significant differences in MPO levels were found in all pairs of research groups (*P* < 0.05). One study by Liu *et al.* ^17^, investigated the role of MPO in RIRI and found that MPO activity increased significantly in rat kidney tissue that experienced RIRI. The results obtained in this study are similar to previous studies that showed that administration of metamizole can reduce kidney necrosis levels in RIRI cases^17^. In this study where low MPO levels were found after being given M100 compared to the negative control group where it can be explained through a study by Unsal and colleagues in 2018 who found that rats that underwent laparotomy had an average increase in MPO up to twice as much as rats that did not undergo laparotomy. This is reinforced by Kazmierski *et al.* ^18^ in 2022 who found that increased postoperative MPO concentrations can be associated with the body’s antioxidant capacity.

In this study, histopathology assessment was performed using the EGTI scoring system, where there was a significant difference in cumulative EGTI scores among the study groups with the lowest value in the negative control and the highest value in the positive control. Meanwhile, the administration of M100 and M200 did not have a significant difference in EGTI scoring. In ischaemic conditions, vascular changes were found where in the first two hours a decreased endothelial function could be seen. After reperfusion occurred, some peritubular capillaries could function again. However, in some other capillaries necrosis will occur and cause chronic ischaemia, where this condition will progress further and cause injury to the tubular and epithelial cells^19^. Until now there has been no previous study conducted to see the effect of metamizole on kidney tissue. However, another study conducted on rat ovarian tissue showed improvement with fewer dilated blood vessels, milder congestion, and less oedema^20^.

The feasibility to experiment on human studies is still debatable until now, but there are lots of similarities between rat models and humans^21^. While not a perfect match, these models offer valuable insights for several compelling reasons. Rats and humans share numerous genes and physiological processes that play pivotal roles in health and disease. This genetic likeness allows researchers to delve into genetic variations and mutations in rats, shedding light on corresponding mechanisms in humans^21^. Additionally, rats’ physiological similarity in terms of organ systems and processes such as the cardiovascular, nervous, endocrine, and immune systems, provides a valuable platform for understanding disease development and treatment responses that resonate with human conditions^21^. The rapid reproductive cycle of rats accelerates research timelines, enabling observation of disease progression and treatment outcomes in a relatively short period. Moreover, the manageability of rats in laboratory settings, owing to their smaller size, facilitates precise experimental control, enhancing the reliability of findings^21^. Ethically, rat models often align with ethical considerations due to their size and lifespan. While rat models aren’t exact replicas of human biology, their genetic and physiological affinities make them a valuable resource for unravelling disease mechanisms and evaluating potential therapies with implications for human health^21^.

Due to the very novel topic of metamizole on RIRI, our study is at a disadvantage where very limited literature is available to make comparison to. Being an animal experimental study, this finding could only provide initial evidences that requires further investigations. Future clinical trials proceeding through the formal and ethical development process should be done in order to evaluate and conclude the effect of metamizole for RIRI in humans.

Bases on our study we found that metamizole with certain dosage has the potential to provide protection to the kidney by anti-inflammatory mechanisms and reducing oxidative stress caused by ischaemic injury.

## Conclusion

Metamizole administration can reduce IL-18 and MPO levels in RIRI conditions with 100 mg/kgBW, giving more optimal results but not affecting NGAL levels. Metamizole administration can reduce cumulative EGTI scores in RIRI conditions, both at doses of 100 and 200 mg/kgBW. This study shows that Metamizole can be used to prevent kidney injury in RIRI, and IL-18 and MPO can be biomarkers in predicting RIRI occurrence.

## Ethical approval

Research approval was obtained from the Health Research Ethics Committee of Universitas Sumatera Utara Faculty of Medicine with Number 1193/KEP/USU/2021.

## Consent

The present study followed international, national and/or institutional guidelines for humane animal treatment and complied with relevant legislation; (b) the present study involved clientowned animals and demonstrated a high standard (best practice) of veterinary care and involved informed client consent.

## Source of funding

Financial support and sponsorship: none.

## Author contribution

Conceptualization: D.D.K., S.M.W., J.I., E.F., I.A. Data curation: K.R., M.I. Formal analysis: D.D.K., K.R., M.I., I.A. Investigation: D.D.K., K.R., E.F. Methodology: D.D.K., S.M.W., J.I. Project administration: J.I., E.F., J.H., I.A. Resources: S.M.W., J.I. Software: D.D.K., K.R., M.I., J.H. Supervision: S.M.W., J.I., E.F., I.A. Validation: S.M.W., J.I., E.F., J.H. Writing—original draft: D.D.K., K.R., M.I. Writing—review and editing: S.M.W., J.I., E.F., J.H., I.A.

## Conflicts of interest disclosure

None.

## Research registration unique identifying number (UIN)

This study does not involve patients in the experimental study.

## Guarantor

Dhirajaya Dharma Kadar.

## Data availability statement

The data that support the findings of this study are available on request from the corresponding author.

## Provenance and peer review

Not commissioned, externally peer-reviewed.

## References

[1] Shiva N Sharma N Kulkarni YA. Renal ischemia/reperfusion injury: an insight on in vitro and in vivo models. Life Sci 2020;256:117860. 32534037 10.1016/j.lfs.2020.117860

[2] Malek M Nematbakhsh M. Renal ischemia/reperfusion injury; from pathophysiology to treatment. J Ren Inj Prev 2015;4:20–27. 26060833 10.12861/jrip.2015.06 PMC4459724

[3] Edelstein CL. Biomarkers of acute kidney injury. Adv Chronic Kidney Dis 2008;15:222–234. 18565474 10.1053/j.ackd.2008.04.003 PMC3287955

[4] Van Biesen W Vanholder R Lameire N. Defining acute renal failure: RIFLE and beyond. Clin J Am Soc Nephrol 2006;1:1314–1319. 17699363 10.2215/CJN.02070606

[5] Wu H Craft ML Wang P. IL-18 contributes to renal damage after ischemia-reperfusion. J Am Soc Nephrol 2008;19:2331–2341. 18815244 10.1681/ASN.2008020170 PMC2588109

[6] Devarajan P. Update on mechanisms of ischemic acute kidney injury. J Am Soc Nephrol 2006;17:1503–1520. 16707563 10.1681/ASN.2006010017

[7] Munshi R Johnson A Siew ED. MCP-1 gene activation marks acute kidney injury. J Am Soc Nephrol 2011;22:165–175. 21071523 10.1681/ASN.2010060641 PMC3014045

[8] Noto A Cibecchini F Fanos V. NGAL and metabolomics: the single biomarker to reveal the metabolome alterations in kidney injury. BioMed Res Int 2013;2013:1–6. 10.1155/2013/612032 PMC3625560 23607092

[9] Khan A Alsahli M Rahmani A. Myeloperoxidase as an active disease biomarker: recent biochemical and pathological perspectives. Med Sci (Basel) 2018;6:33. 29669993 10.3390/medsci6020033 PMC6024665

[10] Khalid U Pino-Chavez G Nesargikar P. Kidney ischaemia reperfusion injury in the rat: the EGTI scoring system as a valid and reliable tool for histological assessment. J Histol Histopathol 2016;3:1.

[11] Bachmann F Duthaler U Rudin D. N-demethylation of N-methyl-4-aminoantipyrine, the main metabolite of metamizole. Eur J Pharm Sci 2018;120:172–180. 29746911 10.1016/j.ejps.2018.05.003

[12] Sánchez S Martín MJ Ortiz P. Effects of dipyrone on inflammatory infiltration and oxidative metabolism in gastric mucosa: Comparison with acetaminophen and diclofenac. Dig Dis Sci 2002;47:1389–1398. 12064817 10.1023/a:1015395103160

[13] Kilkenny C Browne WJ Cuthill IC. Improving Bioscience Research Reporting: The ARRIVE Guidelines for Reporting Animal Research. PLoS Biol 2010;8:e1000412. 20613859 10.1371/journal.pbio.1000412 PMC2893951

[14] Keseroglu BB Ozgur BC Surer H. Which agent should be used to reduce ischemia-reperfusion injury after testicular torsion: a comparative animal experiment. J Pediatr Urol 2019;15:607.e1–607.e7. 10.1016/j.jpurol.2019.06.005 31288984

[15] Schulte B Tergast TL Griemsmann M. Metamizole-associated risks in decompensated hepatic cirrhosis [Internet]. Dtsch Arztebl Int 2022;119:687–693. 35912424 10.3238/arztebl.m2022.0280 PMC9830680

[16] Mårtensson J Bellomo R. The rise and fall of NGAL in acute kidney injury. Blood Purif 2014;37:304–310. 25170751 10.1159/000364937

[17] Liu Z Yang Q Wei Q. The protective effect of miR-377 inhibitor against renal ischemia-reperfusion injury through inhibition of inflammation and oxidative stress via a VEGF-dependent mechanism in mice. Mol Immunol 2019;106:153–158. 30612004 10.1016/j.molimm.2018.12.028

[18] Kaźmierski J Miler P Pawlak A. Increased postoperative myeloperoxidase concentration associated with low baseline antioxidant capacity as the risk factor of delirium after cardiac surgery. Ann Med 2022;54:610–616. 35175161 10.1080/07853890.2022.2039405 PMC8856092

[19] Basile DP. The endothelial cell in ischemic acute kidney injury: implications for acute and chronic function. Kidney Int 2007;72:151–156. 17495858 10.1038/sj.ki.5002312

[20] Kumbasar S Salman S Al R. The effect of metamizole on ischemia/reperfusion injury in the rat ovary: an analysis of biochemistry, molecular gene expression, and histopathology. Indian J Pharmacol 2016;48:32. 26997719 10.4103/0253-7613.174515 PMC4778203

[21] Szpirer C. Rat models of human diseases and related phenotypes: a systematic inventory of the causative genes. J Biomed Sci 2020;27:84. 32741357 10.1186/s12929-020-00673-8 PMC7395987

